# Discriminating Potential Genetic Markers for Complete Response and Non-Complete Response Patients to Neoadjuvant Chemotherapy with Locally Advanced Rectal Cancer

**DOI:** 10.3390/ijerph19074008

**Published:** 2022-03-28

**Authors:** Jaya Bagaria, Kyung-Ok Kim, Eva Bagyinszky, Seong Soo A. An, Jeong-Heum Baek

**Affiliations:** 1 Department of Bionano Technology, Gachon University, Seongnam-si 13120, Korea; jbagaria1206@gmail.com (J.B.); navigator120@gmail.com (E.B.); 2 Gachon Medical Research Institute, Gil Medical Center, Gachon University, Incheon 21565, Korea; kokim67@gachon.ac.kr; 3 Division of Colon and Rectal Surgery, Department of Surgery, Gil Medical Center, College of Medicine, Gachon University, Incheon 21565, Korea

**Keywords:** rectal cancer, genetic biomarker, whole exome sequencing, bioinformatic analysis

## Abstract

Background: Neoadjuvant chemoradiotherapy (nCRT) prior to surgery is considered standard therapy for locally advanced rectal cancer. Unfortunately, most patients with rectal cancer are resistant to radiotherapy. This might be a genetic cause. The role of certain rectal cancer-causing genes has not been completely elucidated. This study aims to investigate the genes responsible for locally advanced rectal cancer patients not reacting to radiotherapy. Methods: Whole exome sequencing of the DNA samples was performed on the samples. Bioinformatic analysis on the subjects was established. Individual genetic information was screened to identify differently expressed genes that more frequently appeared in non-complete response (NCR) compared to complete response (CR) patients after nCRT. All variations were verified by Sanger sequencing. Results: Genotyping information and pathway analyses of the samples indicated genes such as FLCN, CALML5, and ANTXR1 to be commonly mutated in CR group, whereas genes such as GALNTL14, CNKSR1, ACD, and CUL3 were more commonly mutated in the NCR group. Chi-square test revealed some significant variants (<0.05) such as rs3744124 (FLCN), rs28365986 (ANTXR1), rs10904516 (CALML5), rs3738952 (CUL3), rs13394 and rs2293013 (PIH1D1), rs2274531 (GPA33), rs4963048 (BRSK2), rs17883366 (IL3RA), rs2297575 (PSMD5), rs2288101 (GALNT14), and rs11954652 (DCTN4). Conclusion: Identifying an array of genes that separate NCRs from CRs would lead to finding genetic biomarkers for early detection of rectal cancer patients that are resistant to nCRT. A further investigation to validate the significance of genetic biomarkers to segregate NCRs from CRs should be performed with a larger CRC dataset. Protein expression levels, as well as transcriptomic analysis, would also help us understand the mechanism of how these genes could play a role in preventing radiation therapy to patients. This would be essential to prevent redundant radiation therapy.

## 1. Introduction

Colorectal cancer (CRC) is one of the most common cancers in the world, which has the third highest incidence and the second ranked mortality rate [1]. CRC has the second highest prevalence in South Korea with 44.5 new cases per 100,000 persons occurring in 2018 [2]. Neoadjuvant chemoradiotherapy (nCRT) followed by total mesorectal excision is considered standard therapy for locally advanced rectal cancer, which can reduce the toxicity of radiation and locoregional recurrence rate compared to postoperative CRT [3,4]. Furthermore, pathologic complete response (pCR) after nCRT in rectal cancer improves oncologic outcomes compared to non-response to nCRT. Therefore, it is essential to predict the efficacy of nCRT for rectal cancer in advance to assist in treatment decisions. Some biomarkers were suggested to predict the tumor response after nCRT in rectal cancer [5]. Even though predicting response to chemoradiotherapy is clinically important, robust biomarkers that can predict the response of rectal cancer to nCRT [6,7] are not known. Moreover, there are no currently decisive biomarkers through genetic analysis using blood to predict a pathological complete response or no response to nCRT in rectal cancer.

Less than 5% of all CRC cases have a causative genetic variability. CRC is an extremely heterogeneous disease with no genetic biomarkers to detect tumor or determine prognosis and treatment response [8]. Among all CRC cases, 6% have a genetic heritability [9]. One in every 20 people develop CRC, either sporadic or inherited. Sporadic cases of CRC are mostly due to dietary habits and environmental factors such as aging. A germline mutation of the DNA in a cell may be transmitted from parents which may be responsible for inherited cancer. However, a somatic mutation may result in clonal production of the defected cell, producing multiple somatic mutations in the cell. Human cancer often belongs to either of the three commonly mutated genes: tumor suppressor genes, oncogenes, and mismatch repair genes [10,11]. Tumor suppressor genes are normal genes which regulate cell proliferation; however, malignancies may result if these genes are inactivated [12,13]. Mutation in a normal cell, when proliferated uncontrollably, becomes an oncogene [12,14]. DNA mismatch repair genes accumulate errors in DNA throughout the genome that affect the growth regulation genes [10,15].

Genetics play an important part in the predisposition of CRC. Most CRC related genes are somatic and appear to occur in a predictable manner. APC (adenomatous polyposis coli) and TP53 mutations, which are tumor suppressor genes, generally occur in the beginning and the late phases, respectively. CRC is caused by many high and low penetrant mutations and risk gene modifiers—such as APC, MLH1, MSH2, MSH6, PMS2, AXIN2, POLD, MYH, KRAS, BRAF, NRAS, CTNNB1, TLR2, and FLCN to name a few [16,17,18,19,20,21]. APC is a tumor-suppressor gene involved in cellular processes such as apoptosis, cell migration, DNA repair. APC mutation may play a pivotal role in early development of CRC by activating Wnt signaling pathways [22]. APC mutations have been observed in more than 90% of CRC patients [23]. TP53, also a tumor-suppressor gene, is often lost in CRC. Loss of function in TP53 contributes to damaged DNA in daughter cells, which has been reported in over 70% of CRC cases indicating a possible carcinoma [24,25,26]. Among all CRC cases, 40% are predisposed by the KRAS gene. Mutations in KRAS lead to EGFR (epidermal growth factor receptor) which is an integral pathway associated with CRC. The expression of EGFR, although not an independent prognostic marker for CRC, has been shown to be significantly associated with tumor progression.

### 1.1. Biomarker Discovery for the Prediction of Tumor Response to Neoadjuvant Chemoradiotherapy in Locally Advanced Rectal Cancer

nCRT prior to surgery is considered standard therapy for locally advanced rectal cancer. It can reduce the locoregional recurrence rate and toxicity of radiation compared to postoperative CRT. Tumor regression, including pathologic complete response after nCRT, is associated with good oncologic outcomes compared to non-response to nCRT. Despite the clinical importance of predicting a response, the response of patients with locally advanced rectal cancer to nCRT varies and has not been predicted. It is crucial to predict the efficacy of nCRT for rectal cancer patients in advance. However, there are no currently robust biomarkers for the prediction of a pathological complete response, and thus it remains an essential issue. Thus, DNA of the cancer samples were sequenced and screened for germline mutations in genes that were more commonly mutated in CR and NCR patients. 

### 1.2. Patients

In this study, 29 rectal cancer patients were selected for whole exome analysis. Among the patients, 14 of them fully reacted to radiotherapy (CR), and 15 of them did not react to radiotherapy (NCR). The absence of viable cancer cells in the tissue is described as a CR. Otherwise, it is said to be NCR. In our study, to clearly see the difference between the two groups, the NCR group was included in the case of stage III in both the preoperative clinical stage and the postoperative histologic stage. The majority of patients were male patients (23), and most patients were in their 50s or 60s. Table 1 summarizes the demographic properties of patients involved in this study. The study was approved by the institutional review board of Gil Medical Center (approval no. GCIRB2013-223).

## 2. Materials and Methods

DNA was extracted from the white blood cells of patients, whole exome sequencing (WES) was performed by Novogene Inc. (Hong Kong, https://en.novogene.com, accessed on 17 February 2022). Measures of 2 µg of DNA were used for WES analysis. Genome analysis was carried out by Illumina HiSeq 2500 sequencing tool. Quality control was performed on the samples, followed by exome and library preparation. After additional QC, samples were sequenced. Detailed bioinformatics analysis was performed on the samples. Data were received as a .bam file, which could be visualized through the IGV tool. Fully annotated files were also sent on INDELs and SNPs. Variants were screened against different reference databases (such as 1000 Genomes or ExAC), and bioinformatic predictions were also performed on the different missense mutations. The patients’ genetic information was individually screened for genes that appeared more commonly in NCR than CR and vice versa. For analysis, we searched for genes in CR and NCR groups, which mutated in only CR or NCR group, but not in the other group. We also searched for genes which were mutated more commonly either in CR or NCR group. All variations were verified by Sanger sequencing.

Pathway analyses were performed on patients with STRING and ClueGO tools. To investigate their potential role in cancers, we added several known cancer-related genes, to suggest how these selected genes could play a role in cancer related pathways. We selected TP53, KRAS, PTEN, MSH2, BRCA1, BRCA2, MUTYH, and APC genes.

**Figure 1:**
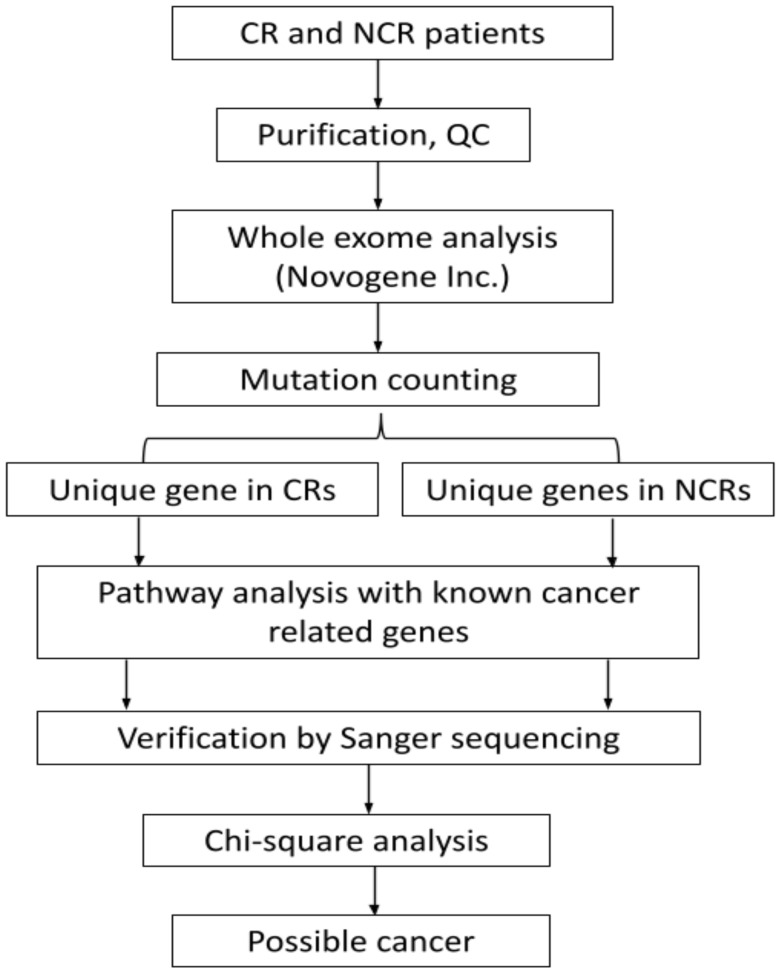
Flowchart of the analysis of commonly mutated genes in CR and NCR.

Statistical analysis was employed by a Chi-square test using SPSS software. Individual variants were tested for chi-square to find the significant (*p* < 0.05) genetic biomarker for each CR or NCR group. The workflow of the methods has been shown in the Figure 1 below. 

## 3. Results

No genes were found, which were only mutated in CR groups, but not in NCRs and vice versa. Two genes, USP19 and RPUSD3, were only mutated in six and five CR patients, respectively, but none of them were mutated in the NCR group. Among NCRs, more uniquely mutated genes were found which were not observed in CRs. Six patients carried mutations for OR5L1, MRM1, and GALNT14 genes. Five patients carried mutations for THEMIS, SLC5A11, PTPRF, OR5L2, MED12L, KRTAP19-8, KNOP1, HIP1, and DAZL genes. In terms of more frequently mutated genes, 23 and 38 genes were observed in the CR and NCR groups, respectively (Table 2).

### 3.1. NCR Genes and Their Association with Rectal Cancer Related Genes through Pathway Analysis

**Figure 2:**
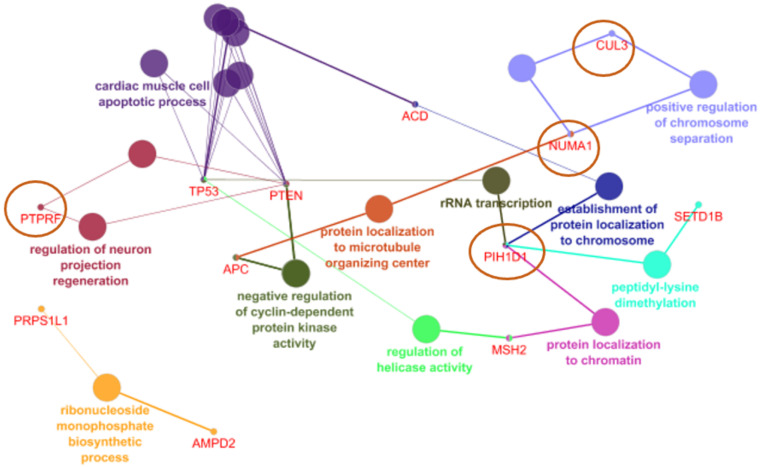
NCR genes and their association with rectal cancer related genes in CLUEGO.

ClueGo analysis revealed six common NCR genes shown in Figure 2, which could be related with known cancer genes: ACD can be related with TP53 through cardiac muscle cell apoptosis. PIH1D1 may also be related to TP53 through rRNA transcription. With the PTEN gene, PTPRF may be associated through regulation of neuron projection. NUMA1 could be related to CUL3 through positive regulation of chromosome regulation and with APC through negative regulation of cyclin dependent protein kinase activity.

**Figure 3:**
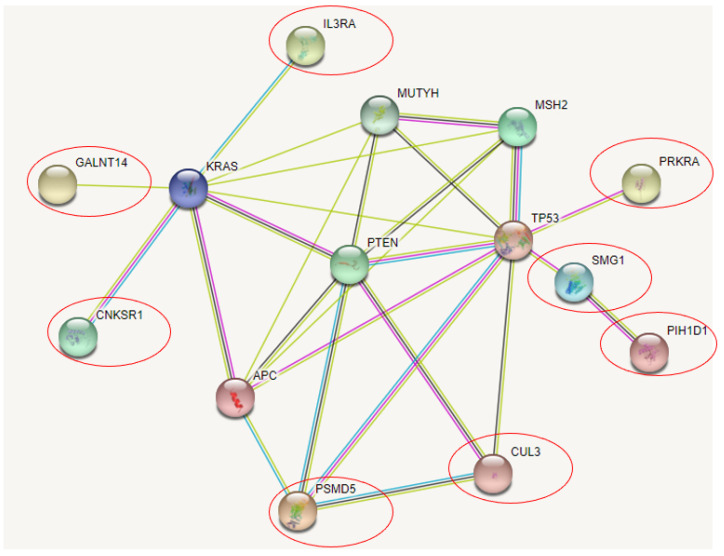
NCR genes and their association with CRC related genes in STRING.

STRING analyzes different genes to be related directly or indirectly with cancer risk genes, shown in Figure 3: GALNT14, CNKSR1, IL3RA, SMG1, PRKRA, and PSMD5. Similarly, with ClueGo, CUL3 and PIH1D1 were also included in the STRING prediction. All over, PTEN was suggested to play a central role in the gene interaction, the NCR-common genes could be associated with it either directly or indirectly. GALNT14, CNKSR1, and IL3RA could be related with KRAS. SMG1 and PRKRA may be associated with TP53. PIH1D4 was predicted to be indirectly associated with TP53 through SMG1. CUL3 was predicted to interact with PTEN and PSMD5. Besides CUL3, PSMD5 may interact directly with APC and PTEN.

### 3.2. CR Genes and Their Association with Rectal Cancer Related Genes through Pathway Analysis

**Figure 4:**
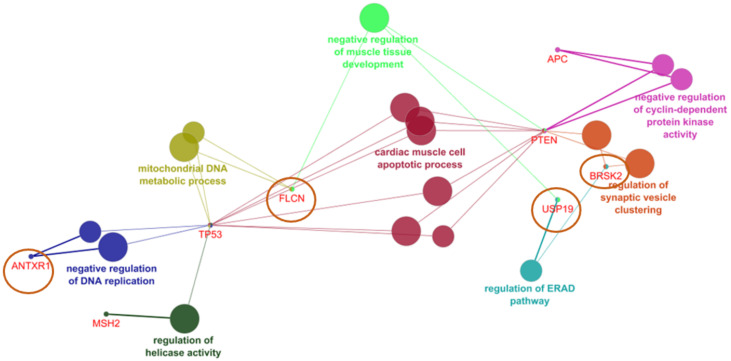
CR genes and their association with rectal cancer related genes in ClueGO.

ClueGo revealed four genes shown in Figure 4, which could associate with the known cancer risk genes: ANTRX1, FLCN, USP19, and BRSK2. ANTRX1 could interact with TP53 and play a role in negative regulation of DNA replication. FLCN could also be associated with TP53 through mitochondrial metabolism. FLCN may also interact with PTEN and involved in the negative regulation of muscle development. BRSK and USP19 could work together in endoplasmic reticulum associated protein degradation, while USP19 may play a role in dysregulation of muscle development. BRSK and PTEN may act together in regulation of synaptic vesicle clustering.

**Figure 5:**
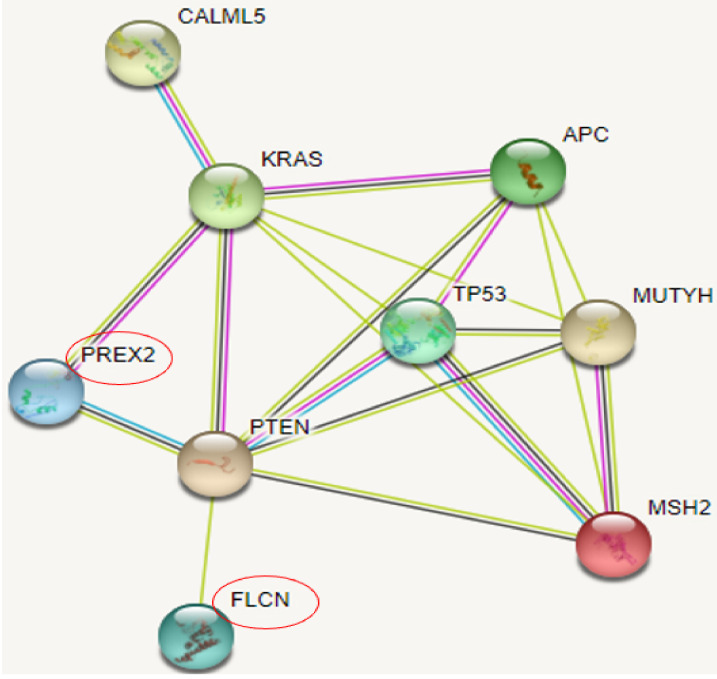
CR genes and their association with rectal cancer related genes in STRING.

STRING revealed three possible genes, shown in Figure 5, associated with known risk genes. PREX2 may present the strongest association among them since they may interact with KRAS and PTEN genes. FLCN could weakly interact with PTEN, while CALML5 was shown to interact with the KRAS gene.

### 3.3. SNPs in the Genes, Found in CR and NCR Groups

The variant-wise analysis revealed 13 and 33 variants in CR and NCR genes, respectively. Among them, 5 and 9 significantly different variants were observed in CRs and NCRs, respectively (Table 3). Among the CRs, FLCN, ANTXR1, and CALML5 carried a variant, which was observed more frequently in CRs, compared to NCRs. Among NCR genes, except for PTPRF and NUMA1, all genes carried at least one variant, which occurred more frequently among NCRs, compared to CRs. In addition, PIH1D1 carried three variants (rs13394, rs2293013, and rs2293012) which were more common in NCRs, compared to CRs. PRKRA carried two variants (rs77419724 and rs9406386), which occurred in seven NCRs, but only two CRs.

## 4. Discussion

In this study, we compared the mutation degrees of different genes among rectal cancer patients, who fully reacted or not reacted to radiotherapy (CR and NCR). Pathway analyses were performed on several genes which were suggested to be associated with CRC. Variants in the selected genes were checked, and mutations were also compared in CR and NCR patients. Mutations which more commonly occurred among CRs and NCRs compared to the other groups were considered further. In CR and NCR patients, 23 and 38 genes were observed (respectively), which were more commonly mutated compared to the other group. Among them, ClueGo and STRING revealed 6 CR and 14 NCR genes, which were associated with the known cancer genes. Variants in these genes were screened: 4 and 13 mutations were found which occurred more commonly in CR and NCR group, respectively. These variants may be possible markers, which could be specific to either CR or NCR rectal cancer groups.

In the CR group, three variants were found in three different genes, which may be significantly higher in CRs. FLCN gene encodes the folliculin protein, which could act as a tumor suppressor, and its germline or somatic mutation may be associated with different kinds of cancers, such as fibrofolliculomas, lung cysts, renal tumors, or renal neoplasia in Birt–Hogg–Dubé (BHD) syndrome. FLCN may be involved in TGF-beta signaling [27]. In addition, FLCN could be involved in the mTOR pathway and may play a role in mitochondrial biogenesis [28]. Mutations (somatic frameshift) in FLCN may also contribute to CRC [29]. Significance of rs3744124 remained unclear, but PolyPhen predictions suggested that the affected residue (G303) may not be conserved among vertebrates [30]. ANTXR1 has an oncogenic function, since it could induce the cell migration, invasion, proliferation, and adhesion. ANTXR1 was verified to be lowly expressed in normal tissues, but its expression increased in cancer tissues, including in individuals with gastric cancer [31,32]. ANTXR1 was suggested to enhance the PI3K/AKT/mTOR pathway. Overexpression of ANTXR1 in gastric cancer patients was associated with poor prognosis [30]. CALML5 is a calmodulin-like skin protein, which plays a role in calcium binding. Ubiquitination of CALML5 may be involved in breast cancer, but its role in other cancers was not investigated yet [33].

Among NCR group, 13 variants were found in nine genes, which occurred in significantly higher number among NCRs. GALNTL14 belongs to the N-acetylgalactosaminyltransferase enzyme family. Abnormal functions of GALNTL family results in aberrant glycosylation pattern, which could be strongly involved in carcinogenesis. GALNTL14 could be associated with different kinds of cancers, including lung and pancreatic carcinomas, or melanomas. In addition, it may impact the resistance of chemotherapy in breast cancer [34]. GALNTL14 variants may impact the response of patients to treatments. For example, rs9679162 GG genotype was suggested to be associated with longer time to respond to chemo- and radiotherapy in esophageal cancer patients [35]. IL3RA is a receptor for IL3 and has a dual role in immune system. It could bind several ligands, and may play a role in cytokine signaling, and could induce or block cancer related mechanisms. Development of drugs, which could target the cytokine receptors, may be involved in cancers [36]. PSMD5 is involved in protein degradation by ubiquitin system. Inactivation of PSMD5 may play a significant role in colorectal tumors by the assembly of 26S proteasome [37]. PRKRA is an activator of PRKR kinase, and its knockout may result in enhanced sensitivity for chemotherapy (oxaliplatin) mouse ovarian cancer cells [38]. CNKSR encodes the kinase suppressor of RAS and could act as oncogene in RAS dependent cancers [39]. CNKSR1 expression was correlated with the survival rate in pancreatic tumor patients and suggested to be an independent prognostic marker for survival. The expression of pERK was also correlated with CNKSR1 distribution (expression of the scaffold connector enhancer of kinase suppressor of Ras 1 (CNKSR1) is correlated with clinical outcome in pancreatic cancer) [40]. ACD could be involved in the proper functions of telomerases. Somatic mutations in ACD may result in imbalance in telomere homeostasis and apoptosis. CUL3 is a ubiquitin ligase, involved in different diseases, such as muscle and metabolic dysfunctions, but also in cancers. CUL3 may be a multifunctional protein which could play a role in different diseases, such as protein trafficking and cell cycle regulation [41]. Mutations in CUL3 and abnormalities in its expression may result in either oncogenic or tumor suppressive processes and could be possible target for treatment strategies [42]. The variant rs3738952 was screened in Chinese lung carcinoma patients, but it remained unclear whether it could result in any dysfunctions [43]. PIH1D1 may impact the oncogenesis and treatment reaction [44]. It could interact with the MTOR complex and is overexpressed in breast cancer [45]. SMG1 is a phosphoinositide 3-kinase-related kinase, involved in nonsense-mediated mRNA decay. It may act as tumor suppressor, especially in hypoxic tumors. SMG1 may be downregulated in AML patients and restoring SMG1 expression could inhibit the AML cell growth. SMG1 expression may also correlate with the MTOR complex and may react antagonistically to AML growth [46]. Overexpression of SMG1 may also play a role in CRC through microsatellite instability [47].

To find out the association among the filtered genes, we performed chi-square test on all the genes except the ones that were exclusively found in NCRs and CRs, such as USP19, RPUSD3, OR5L1, MRM1, GALNT14, THEMIS, SLC5A11, PTPRF, OR5L2, MED12L, KRTAP19-8, and KNOP1. Some of the significant variants that yielded chi-square value of less than 0.05 are (Table 3): rs3744124 (FLCN), rs28365986 (ANTXR1), rs10904516 (CALML5), rs4963048 (BRSK2), rs11954652 (DCTN4), rs2288101 (GALNT14), rs17883366 (IL3RA), rs2297575 (PSMD5), rs3738952 (CUL3), rs13394 (P1H1D1), rs2293013 (P1H1D1), rs2293012 (P1H1D1), rs2274531 (GPA33), and rs28362581 (AMPD). The limitation of this study was the small sample size since only 14 CRs and 15 NCRs were screened. These data should be verified in a larger group of patients in the future and compared with cancer-free populations to validate the significance before applying it to a clinical setting. More genetic screening strategies to find the difference between CR and NCR might contribute to advanced treatment and mortality decline. Furthermore, proteomics and transcriptomics analyses would provide significant insight into the mechanism of why some patients benefit with nCRT and some do not. 

## 5. Conclusions

In this study, we examined CR and NCR patients after nCRT for rectal cancer and performed genetic profiling on them. Our goal was to find commonly mutated genes in CR and NCR groups, which may work as a potential marker on them to reduce redundant chemo-radiation therapy. We also made association studies by pathway analyses, whether these genes could be related to the known cancer causing or risk genes. Pathway analyses found candidates, which may be common in CR, such as FLCN, CALML5, or ANTXR1. In the NCR group, several commonly mutated genes were found too, including GALNTL14, CNKSR1, ACD, or CUL3. These genes may interact with cancer genes, suggesting them as potential risk modifiers in disease progression. Although clinical implications of the genetic difference are not well understood, genetic profile differences of CR and NCR patients may be helpful in cancer treatment prediction.

## Figures and Tables

**Table 1:** Demography of patients with rectal cancer selected for whole exome sequencing.

Patient Info		Number
Reaction to radiotherapy	CR	14
	NCR	15
Gender	Male	23
	Female	6
Age group (Years)	Under 40	1
	40–49	2
	50–59	9
	60–69	8
	70–79	4
	Over 80	5

**Table 2:** List of unique or more frequently mutated genes in CR and NCR groups.

**Unique Genes in CRs**
**Only occurred in CRs:** USP19, RPUSD3
**More commonly mutated in CRs:** PREX2, IRGM, TTLL3, HELT, FLCN, EDDM3B, WARS2, PDE4A, KIAA0895L, GLIS1, OR11A1, DCAF4, DHFRL1, C17orf96, ALDH5A1, ANTXR1, ACBD5, ELF1, DCTN4, NAV1, RAI14, CALML5, BRSK2
**Unique Genes in NCRs**
**Only occurred in NCRs:** OR5L1, MRM1, GALNT14, THEMIS, SLC5A11, PTPRF, OR5L2, MED12L, KRTAP19-8, KNOP1, HIP1, DAZL
**More commonly mutated in NCRs:** IL3RA, AMER2, GPA33, ZNF546, PSMD5, CLEC3A, ZNF552, YDJC, SMG1, SAMD7, NCBP3, HPS1, TIMM21, RBP1, SLC4A1AP, PRPS1L1, PSG8, PLSCR4, PRKRA, LRRC8B, LY6G5B, OR5I1, OR7D4, NUMA1, FILIP1L, ERC1, CPD, C2orf61, CD8B, CNKSR1, AVL9, ACD, FAAH, AMPD2, CUL3, PIH1D1, FCRL3

**Table 3:** Occurrence of variants in in CR and NCR genes.

Group	Gene	SNP	AA Change	GNOMAD_All	GNOMAD_EAS	NCBI-KR	SIFT	PPH2	CR	NCR	Chi-sq (*p* < 0.05)
**CR**	**USP19**	rs199572044	L305P	0.00002628	0.0007692	0.0017	0.002, D	0.925, P	1	NA	0.483
		rs11552724	D36H	0.07581	0.03814	0.0606	0.042, D	0.919, P	3	NA	0.10
		rs144742940	G59D	0.0008365	0.02230	0.0623	0.448, T	0.207, B	3	NA	0.10
	**PREX2**	rs141504768	V678L	0.0057	0.0823	0.0717	0.124, T	0.044, B	5	2	0.22
		rs61753703	R1394W	0.006858	0.0334	0.0246	0.0, D	1.0, D	2	2	1.0
		rs61753704	S1488L	0.0178	0.1671	0.1832	0.038, D	0.999, D	4	1	0.17
	**FLCN**	**rs3744124**	**G303R**	**0.07370**	**0.1792**	**0.2124**	**0.377, T**	**0.002, B**	**9**	**2**	**0.008**
	**ANTXR1**	**rs28365986**	**R7K**	**0.01144**	**0.1094**	**0.1453**	**0.467, T**	**0.009, B**	**9**	**1**	**0.002**
		NA	Y278H	NA	NA	NA	0.532, T	0.999, D	1	NA	0.483
	**CALML5**	**rs10904516**	**K74R**	**0.3704**	**0.3016**	**0.2906**	**1.0, T**	**0.76, P**	**11**	**4**	**0.009**
		rs11546426	S58G	0.3147	0.2015	0.1939	0.015, D	0.007, B	4	4	1.0
	**BRSK2**	**rs4963048**	**T760A**	**0.688**	**0.4424**	**0.3690**	**1.0, T**	**0.0, B**	**12**	**4**	**0.003**
		rs752637187	A213S	0.00001314	0.0003849	NA	0.247, T	0.993, D	1	NA	0.483
	**DCTN4**	**rs11954652**	**F349L**	**0.2029**	**0.2454**	**0.2290**	**1.0, T**	**0.0, B**	**10**	**3**	**0.009**
**NCR**	**GALNT14**	**rs2288101**	**Q449K**	**0.2141**	**0.2220**	**0.1890**	**0.921, T**	**0.962, D**	**NA**	**5**	**0.042**
		rs188727997	P74L	0.00003286	0.00	NA	0.007, D	0.977, D	NA	1	1.0
	**PTPRF**	rs3748800	D562N	0.000632	0.01612	0.0342	0.434, T	1.0, D	NA	2	0.483
		rs540407495	E1171K	0.00001971	0.0005767	0.00513	0.617, T	0.032, B	NA	1	1.0
		rs368723795	R1207W	0.00003942	0.00	0.00102	0.004, D	0.988, D	NA	1	1.0
		rs17849101	R635C	0.0005256	0.01424	0.01029	0.003, D	1.0, D	NA	1	1.0
	**IL3RA**	**rs17883366**	**V323L**	**0.1410**	**0.09604**	**NA**	**1.0, T**	**0.0, B**	**NA**	**5**	**0.042**
		rs201668157	F281L	0.00005934	0.001553	NA	0.78, T	0.008, B	NA	1	1.0
	**PSMD5**	**rs2297575**	**E21G**	**0.02510**	**0.1991**	**0.1778**	**0.038, D**	**0.749, P**	**2**	**9**	**0.021**
	**PRKRA**	rs77419724	I113N	0.1372	0.0797	0.158	0.013, D	0.996, D	2	7	0.109
		rs9406386	M1L	0.0473	0.0305	0.157	0.0, D	0.0, B	2	7	**0.109**
	**NUMA1**	rs117729282	L344V	0.001064	0.01830	0.0297	0.326, T	0.998, D	NA	2	0.483
		rs3750913	A794G	0.02866	0.0741	0.05631	0.073, T	0.117, B	1	3	0.598
		rs74985106	R1681C	0.01552	0.0159	0.0185	0.003, D	1.0, D	NA	1	1.0
		rs151173629	A96V	0.0001446	0.00	0.002	0.009, D	1.0, D	NA	1	1.0
	**CNKSR1**	rs1045105	H687N	0.1185	0.0883	0.1116	0.658, T	0.0, B	NA	4	0.10
		rs2297710	P350Q	0.0052	0.08190	0.0626	0.267, T	0.971, D	3	4	1.00
	**ACD**	rs6979	V515A	0.5950	0.1533	0.1269	1.0, T	0.0, B	3	7	0.245
	**CUL3**	**rs3738952**	**V567I**	**0.0905**	**0.2657**	**0.3269**	**0.195, T**	**0.0, B**	**3**	**11**	**0.009**
		rs190453078	R46H	0.0000616	0.00	NA	0.175, T	0.996, D	NA	1	1.0
	**PIH1D1**	**rs13394**	**V224I**	**0.7767**	**0.3938**	**0.3288**	**1.0, T**	**0.0, B**	**4**	**11**	**0.027**
		**rs2293013**	**G10E**	**0.6973**	**0.3880**	**0.3286**	**1.0, T**	**0.0, B**	**4**	**11**	**0.027**
		**rs2293012**	**M9L**	**0.6973**	**0.3894**	**0.3286**	**1.0, T**	**0.0, B**	**4**	**12**	**0.009**
	**SMG1**	rs147586756	T1060I	0.00212	0.05399	0.04778	0.026, D	0.037, B	NA	3	0.224
		rs12051350	A35T	0.01738	0.1287	0.0778	0.133, T	0.016, B	1	5	0.169
		rs34960798	Q2730E	0.00001314	0.0003852	0.000342	1.0, T	0.055, B	NA	1	1.0
		rs777224856	A2020T	0.0000065	0.0001923	0.001027	0.335, T	0.996, D	NA	1	1.0
	**GPA33**	**rs2274531**	**D20N**	**0.08856**	**0.1601**	**0.1478**	**1.0, T**	**0.0, B**	**1**	**7**	**0.035**
	**AMPD**	**rs28362581**	**A82T**	**0.1352**	**0.3146**	**0.2447**	**0.004, D**	**0.059, B**	**3**	**10**	**0.025**

Abbreviations: NA, not applicable; T, tolerated; B, benign; D, damaging; AA, amino acid; SNP, single nucleotide polymorphism; EAS, east Asian; Chi-sq, Chi-square. Significant variants are indicated in bold.

## Data Availability

The authors confirm that the data supporting the findings of this study are available withing this article and its supplementary materials.

## Supplementary Materials

The following supporting information can be downloaded at: https://www.mdpi.com/article/10.3390/ijerph19074008/s1.
