# Supplementary material for: Discriminating Potential Genetic Markers for Complete Response and Non-Complete Response Patients to Neoadjuvant Chemotherapy with Locally Advanced Rectal Cancer
Source: Int J Environ Res Public Health. 2022 Mar 28;19(7):4008. doi: 10.3390/ijerph19074008 (PMC8997875; doi:10.3390/ijerph19074008)
Supplement: Supplementary file 1 [file ijerph-19-04008-s001.zip › ijerph-1623594-supplementary.pdf]

Supplementary

Statistically insignificant variants were not verified by Sanger Sequencing.

NUMA1

NUMA1 was found in 7 NCRs (5 male and 2 female) and 1 CR (male) patients. Overexpression of NUMA1 may modulate p53 associated transcription in cancer cells (Endo et al. 2013). NUMA1 expression may be associated with cancer cell survival and resistance to cancer treatment (Gisler et al. 2020).

Variants in NUMA1:

**Gene**	SNP	AA CHANGE	1000g_eas	1000g_all	ExAC_ALL	ExAC_EAS	SIFT	Polyphen 2	NCR number (n/15)	CR number (n/14)
**NUMA1**	rs117729282 (NC_000011.10: g.72017776G>C)	L344V	0.0089	0.00239617	0.002	0.0186	0.326,T	0.998,D	2	NA
	rs3750913 (NC_000011.10: g.72015122G>C)	A794G	0.0833	0.0439297	0.0343	0.0741	0.073,T	0.117,B	3	1
	rs74985106 (NC_000011.10: g.72008984G>A)	R1681C	0.0188	0.0133786	0.0198	0.0167	0.003,D	1.0,D	1	NA
	rs151173629 (NC_000011.10: g.72023069G>A)	A96V	NA	0.000199681	0.0001	0.0006	0.009,D	1.0,D	1	NA

CUL3

CUL3 was found in 3 CRs (male) and 11 NCRs (7 male and 4 female) patients. CUL3 may play a role in both tumor progression and impact the therapeutic response by interacting with other proteins (H.-Y. Chen and Chen 2016).

Variants in CUL3:

**Gene**	SNP	AA CHANGE	1000g_eas	1000g_all	ExAC_ALL	ExAC_EAS	SIFT	Polyphen 2	NCR number (n/15)	CR number (n/14)
**CUL3**	rs3738952 (NC_000002.12:g.224497761C>T)	V567I	0.2391	0.139377	0.1280	0.2772	0.195,T	0.0,B	11	3

	rs190453078 (NC_000002.12:g.224557786C>T)	R46H	.	0.000399361	0.00006687	0	0.175,T	0.996,D	1	NA	

PIH1D1

PIH1D1 was found in 12 NCRs (8 male and 4 female) and 4 CRs (3 male and 1 female) patients. It may impact the oncogenesis and treatment reaction (Kakihara et al. 2020). It could interact with mTOR complex and are overexpressed in breast cancer (Kamano et al. 2013).

Variants in PIH1D1:

**Gene**	SNP	AA CHANGE	1000g_eas	1000g_all	ExAC_ALL	ExAC_EAS	SIFT	Polyphen2	NCR number (n/15)	CR number (n/14)
**PIH1D1**	rs13394 (NC_000019.10:g.49447041C>T)	V224I	0.4177	0.688898	0.7139	0.3985	1.0,T	0.0,B	11	4
	rs2293013 (NC_000019.10:g.49451546C>T)	G10E	0.4117	0.608826	0.6862	0.3870	1.0,T	0.0,B	11	4
	rs2293012 (NC_000019.10:g.49451550T>G)	M9L	0.4117	0.608826	0.6862	0.3867	1.0,T	0.0,B	12	4

Heterozygous for rs2293013 and rs2293012:

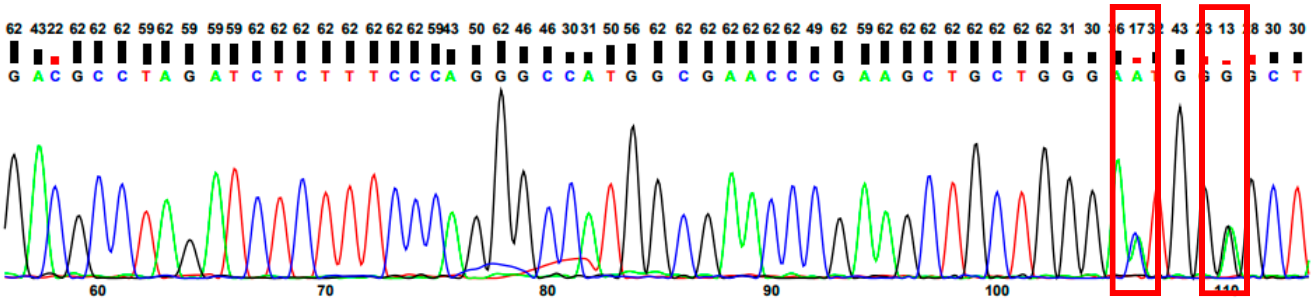

Homozygous for rs2293013 and rs2293012:

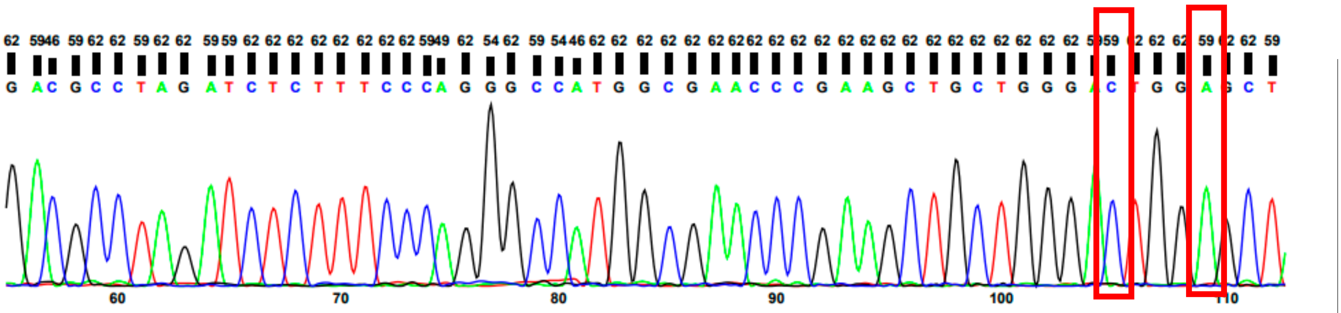

	2G>A)									
**PTPRF**	rs540407495 (NC_000001.11:g.43606267G>A)	E1171K	0.001	0.000199681	0.00009241	0.0013	0.617, T	0.032,B	1	NA
	rs368723795 (NC_000001.11:g.43606375C>T)	R1207W	NA	NA	0.00002471	0.0002	0.004, D	0.988,D	1	NA
	rs17849101 (NC_000001.11:g.43597837C>T)	R635C	0.0119	0.00259585	0.002	0.0235	0.003, D	1.0,D	1	NA

PTPRF

PTPRF was found in 5 NCRs (4 male and 1 female) but not in CRs. PTPRF may act as a tumor suppressor and its expression may be reduced in gastric adenocarcinoma (Tian et al. 2018). It may also help promote the Wnt signaling in CRC (Stevens, Gan, and Gao 2019).

Variants in PTPRF:

GALNT14

GALNT14 was found in 6 NCRs (male) but not in CRs. GALNTs are involved in metastasis in lung and breast cancers (Song et al. 2016). It may act through KRAS/PI3K/c-JUN signaling. Elevated GALNT14 expression could promote metastasis. In CRC, a variant in GALNT14, rs9679162 TT, was associated with poorer survival rate after chemotherapy (Lin et al. 2016).

Variants in GALNT14:

**Gene**	SNP	AA CHANGE	1000g_eas	1000g_all	ExAC_ALL	ExAC_EAS	SIFT	Polyphen2	NCR number (n/15)	CR number (n/14)
**GALNT14**	rs2288101	Q449K	0.1984	0.198283	0.2262	0.2136	0.921, T	0.962,D	5	NA

	(NC_000002.12:g.30912318G>T)									
	rs188727997 (NC_000002.12:g.30992856G>A)	P74L	NA	0.000199681	0.00001652	0	0.007, D	0.977,D	1	NA

Heterozygous for rs2288101:

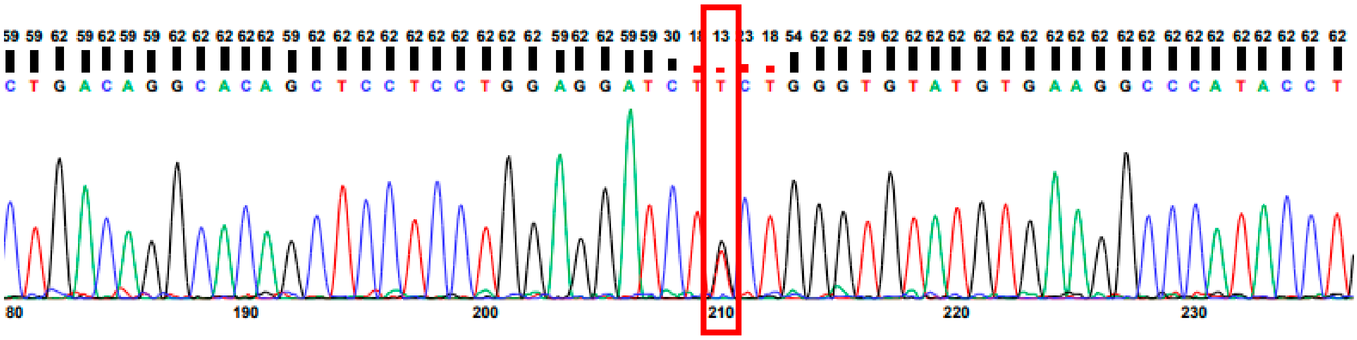

CNKSR1

CNKSR1 was found in 7 NCRs (6 male and 1 female) and 3 CRs (male) patients. It acts as a receptor for tyrosine kinase signaling. CNKSR1 plays a role as scaffold connector and its expression has been said to be correlated with nuclear pERK expression. It may be a potential marker for cancer survival as elevated markers of CNKSR1 could be associated with lower survival (Quadri et al. 2017).

Variants in CNKSR1:

**Gene**	SNP	AA CHANGE	1000g_eas	1000g_all	ExAC_ALL	ExAC_EAS	SIFT	Polyphen2	NCR number (n/15)	CR number (n/14)
**CNKSR1**	rs1045105 (NC_000001.11: g.26189465C>A)	H687N	0.0962	0.118011	0.133	0.0862	0.658,T	0.0,B	4	NA
	rs2297710 (NC_000001.11: g.26184449C>A)	P350Q	0.0903	0.0339457	0.0157	0.0674	0.267,T	0.971,D	4	3

GPA33

GPA33 was found in 7 NCRs (5 male and 2 female) and 1 CR (male) patients. GPA33 has been suggested as a novel receptor for cell surface or adhesion molecule in the family of immunoglobulin (Heath et al. 1997).

Variants in GPA33:

**Gene**	SNP	AA CHANGE	1000g_eas	1000g_all	ExAC_ALL	ExAC_EAS	SIFT	Polyphen2	NCR number (n/15)	CR number (n/14)
**GPA33**	rs2274531 (NC_000001.11: g.167073525C>T)	D20N	0.1726	0.08127	0.0925	0.1606	1.0,T	0.0,B	7	1

Heterozygous for rs2274531:

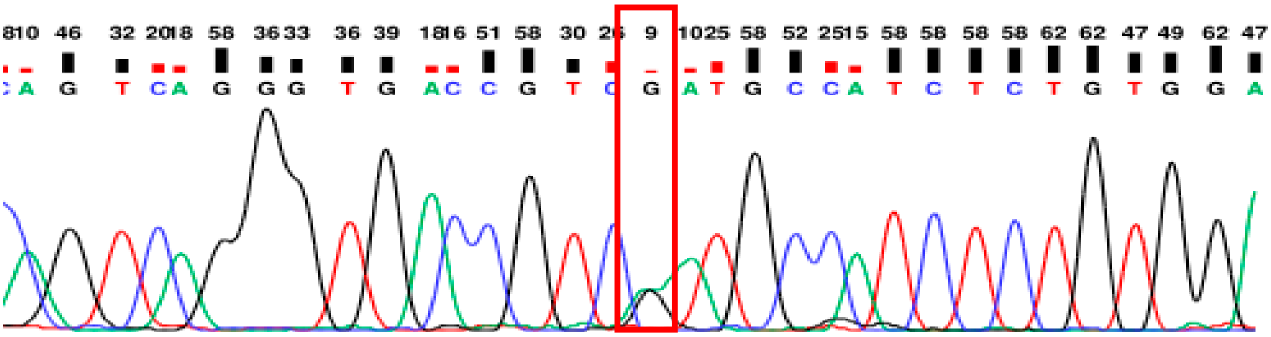

AMPD2

AMPD2 was found in 10 NCRs (8 male and 2 female) and 4 CR (male) patients. AMPD2 is a critical enzyme that catalyzes smooth muscle energy supply and metabolism, however its cellular biology and clinical implication in CRC are largely unknown. The effects of AMPD2 expression on CRC cells and NOTCH3 protein expression were investigated by downregulation and overexpression of AMPD2. AMPD2 may be a novel prognostic biomarker for CRC as it is commonly expressed in CRC and acts as a metabolism oncogene that helps induce CRC progression through the NOTCH signaling pathway (Gao et al. 2020).

Variants in AMPD2:

**Gene**	SNP	AA CHANGE	1000g_eas	1000g_all	ExAC_ALL	ExAC_EAS	SIFT	Polyphen2	NCR number (n/15)	CR number (n/14)
**AMPD2**	rs28362581 (NC_000001.11: g.109621257G>A)	A82T	0.3095	0.236222	0.1345	0.3160	0.004,D	0.059,B	3	10

Heterozygous for rs28362581:

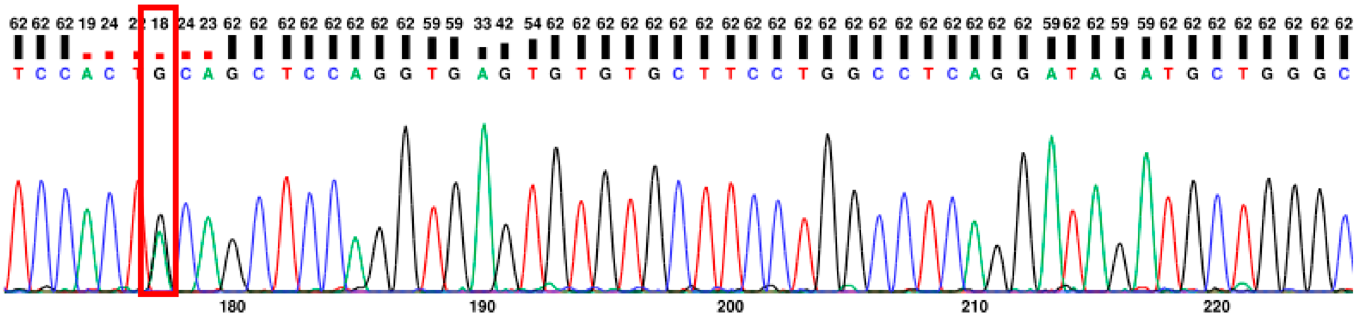

FAAH

FAAH was found in 10 NCRs (8 male and 2 female) and 3 CRs (2 male and 1 female) patients. FAAH blocker PF-3845 effectively decreases viability, migration and invasiveness of the Colo-205 cells at non-cytotoxic concentrations. It was found that pharmacological inhibition of FAAH and consequent enhancement of the endocannabinoid levels may reduce the colorectal cancer growth and progression. These effects have been observed for various FAAH inhibitors in several cancerous cell lines (Wasilewski et al. 2017). In colorectal cancer, FAAH inhibitor AA-5HT (arachidonoyl serotonin) inhibits CaCo-2 cells growth and proliferation, 18 while human colon adenocarcinoma Colo-205 cells show reduced viability, migration, and invasion capabilities after incubation with FAAH inhibitor PF3845 (Maione et al. 2007).

Variants in FAAH:

**Gene**	SNP	AA CHANGE	1000g_eas	1000g_all	ExAC_ALL	ExAC_EAS	SIFT	Polyphen2	NCR number (n/15)	CR number (n/14)
**FAAH**	rs77101686 (NC_000001.11:g.46408574C>T)	A356V	0.0159	0.00838658	0.0034	0.0169	0.003, D	1.0,D	1	NA
	rs75429705 (NC_000001.11:g.46412213C>G)	A476G	0.0169	0.00858626	0.0055	0.0206	1.0,T	0.0,B	1	NA
	rs324420 (NC_000001.11:g.46405089C>A)	P129T	0.1756	0.261581	0.2333	0.1754	0.318, T	0.014,B	9	3

USP19

USP19 was found in 6 CRs (4 male and 2 female) but not in NCR patients. USP19 may function as a tumor suppressor in CRC, as USP19 expression is significantly reduced in CRC tissues based on multiple downloaded datasets. Over-expression of USP19 inhibits CRC

proliferation and migration in-vitro. Conversely, knockdown of USP19 promoted proliferation and migration *in vitro* as well as promoting tumor growth *in vivo* (Hu et al. 2020).

Variants in USP19:

**Gene**	SNP	AA CHANGE	1000g_eas	1000g_all	ExAC_ALL	ExAC_EAS	SIFT	Polyphen2
**USP19**	rs199572044 (NC_000003.12: g.49116909A>G)	L305P	0.001	0.000199681	4.202e-05	0.0006	0.002,D	0.925,P
	rs11552724 (NC_000003.12: g.49119040C>G)	D36H	0.0496	0.053115	0.083	0.0409	0.042,D	0.919,P
	rs144742940 (NC_000003.12: g.49118069C>T)	G59D	0.0198	0.00459265	0.0021	0.0293	0.448,T	0.207,B

DCTN4

DCTN4 was found in 10 CRs (8 male and 2 female) and 3 NCRs (male) patients. Up-regulated DCTN4 in colon adenocarcinoma is correlated with satisfactory prognosis and may be applied as a biomarker for prognosis in CRC patients (Wang et al. 2018).

Variants in DCTN4:

**Gene**	SNP	AA CHANGE	1000g_eas	1000g_all	ExAC_ALL	ExAC_EAS	SIFT	Polyphen2	CR number (n/14)	NCR number (n/15)
**DCTN4**	rs11954652 (NC_000005.10: g.150718321G>C)	F349L	0.2589	0.270767	0.1274	0.2642	1.0,T	0.0,B	10	3
	rs3733923 (NC_000005.10: g.150711219C>T)	S445N	0.0853	0.0185703	0.0075	0.0917	0.445,T	0.0,B	6	NA
	rs141645096 (NC_000005.10: g.150722908C>T)	V310I	0.0149	0.00299521	0.0012	0.0157	0.538,T	0.003,B	1	NA
	rs117873033 (NC_000005.10: g.150718284T>C)	TR362A	0.005	0.000998403	0.0002	0.0031	0.026,D	0.128,B	1	NA

Homozygous for rs11954652:

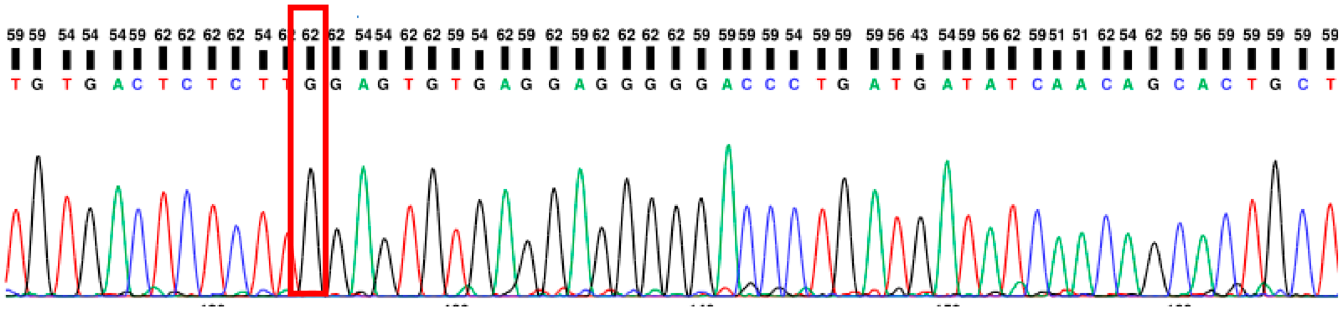

BRSK2

BRSK2 was found in 12 CRs (11 male and 1 female) and 4 CRs (2 male and 2 female) patients. This gene plays a role in neuronal polarization. It is expressed in pancreatic islets (X.-Y. Chen et al. 2012). A mutation in this gene may also be involved in the pancreatic cancer through downregulation of Akt signaling (Saiyin et al. 2017).

Variants in BRSK2:

Gene	SNP	AA CHANGE	1000g_eas	1000g_all	ExAC_ALL	ExAC_EAS	SIFT	Polyphen2	CR number (n/14)	NCR number (n/15)
**BRSK2**	rs4963048 (NC_000011.10: g.1460700A>G)	T760A	NA	NA	0.6096	0.4545	1.0,T	0.0,B	12	4
	rs752637187 (NC_000011.10: g.1443492G>T)	A213S	NA	NA	0.00003796	0.0003	0.247,T	0.993,D	1	NA

Homozygous for rs4963048:

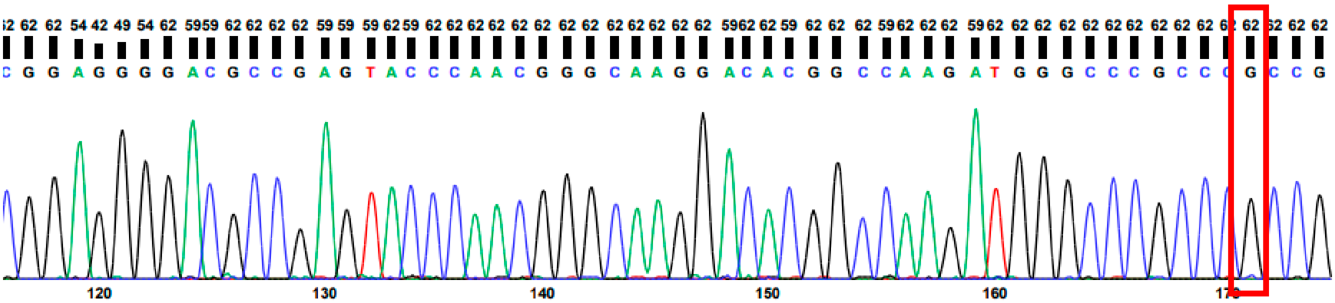

Heterozygous for rs4963048:

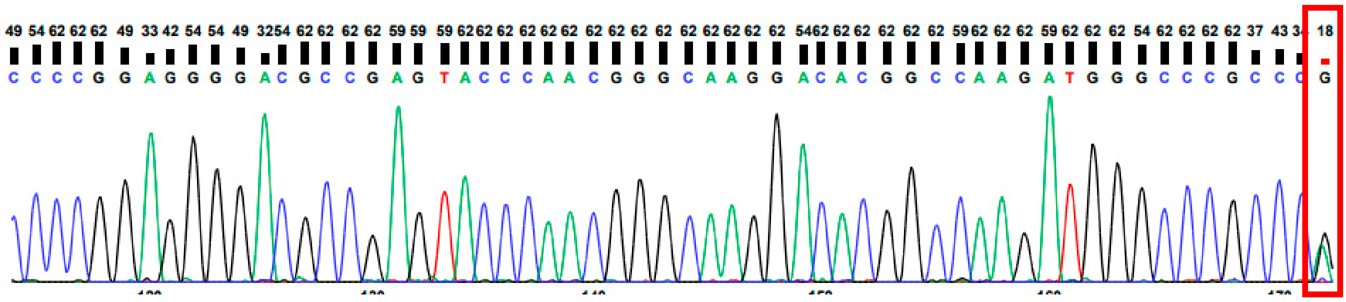

FLCN

FLCN was found in 9 CRs (8 male and 1 female) and 2 NCRs (male) patients. A mutation in FLCN plays a role in endocytosis and cell movement and may also act as a tumor suppressor. This gene is also involved in mTOR activation (Tsun et al. 2013). It also impacts TGF beta activation in renal cancer (Cai et al. 2020). The variant could not be verified by Sanger Sequencing.

Variants in FLCN:

**Gene**	SNP	AA CHANGE	1000g_eas	1000g_all	ExAC_ALL	ExAC_EAS	SIFT	Polyphen2	CR number (n/14)	NCR number (n/15)
**FLCN**	rs3744124 (NC_000017.11: g.17221501C>T)	G303R	0.1488	0.0996406	0.0725	0.1977	0.377,T	0.002,B	9	2

ANTXR1

ANTXR1 was found in 9 CRs (7 male and 2 female) and 1 NCR (male) patients. It is a tumor specific endothelial marker. It plays a role in colorectal cancer and in several oncogenic mechanisms by proliferation and cell cycle progression, invasion and migration and carcinogenicity, and induced suppressed apoptosis. A mutation in this gene may act through PI3K/AKT/mTOR signaling pathways (Cai et al. 2020). Antrax toxin may be used in therapies for cancer (Bachran and Leppla 2016).

Variants in ANTXR1:

**Gene**	SNP	AA CHANGE	1000g_eas	1000g_all	ExAC_ALL	ExAC_EAS	SIFT	Polyphen2	CR number (n/14)	NCR number (n/15)
**ANTXR1**	rs28365986 (NC_000002.12: g.69013519G>A)	R7K	0.1012	0.0355431	0.0366	0.1525	0.467,T	0.009,B	9	1
	NA (NC_000002.12 g.69350178T>C)	Y278H	NA	NA	NA	NA	0.532,T	0.999,D	1	NA

Heterozygous for rs28365986:

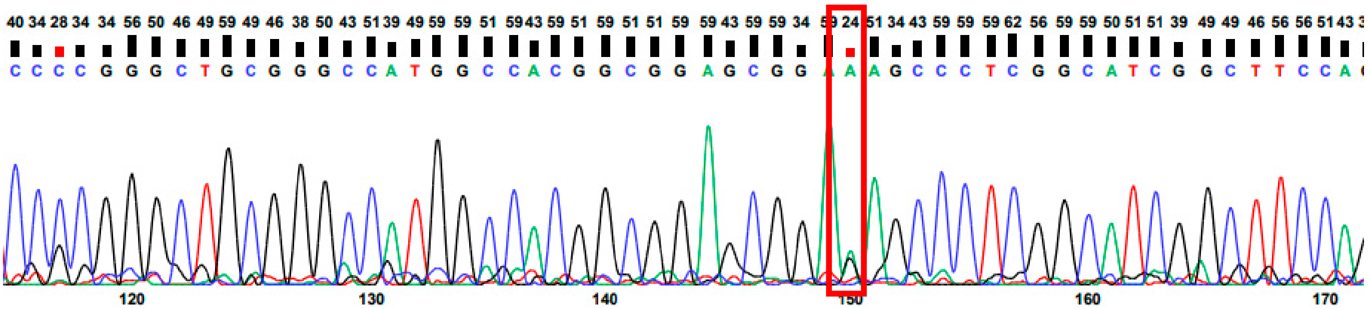

CALML5

CALML5 was found in 11 CRs (9 male and 2 female) and 4 NCRs (3 male and 1 female) patients. Ubiquitination of CALML5 may be involved in carcinogenesis of breast cancer (Debald et al. 2013). However, role of CALML5 in CRC remains unclear.

Variants in CALML5:

**Gene**	SNP	AA CHANGE	1000g_eas	1000g_all	ExAC_ALL	ExAC_EAS	SIFT	Polyphen2	CR number (n/14)	NCR number (n/15)
**CALML5**	rs10904516 (NC_000010.10: g.5541181T>C)	K74R	0.3046	0.353235	0.3348	0.3007	1.0,T	0.76,P	11	4
	rs11546426 (NC_000010.11: g.5499267T>C)	S58G	0.2014	0.296925	0.2852	0.2062	0.015,D	0.007,B	4	4

Heterozygous for rs10904516:

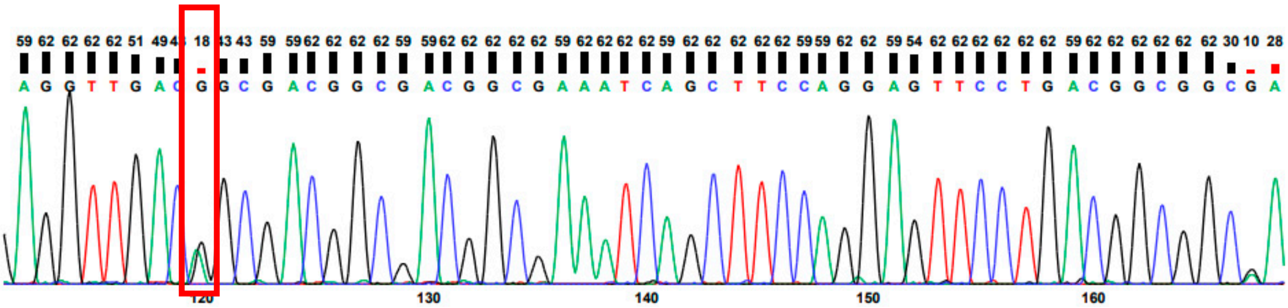

PREX2

PREX2 was found in 9 CRs (8 male and 1 female) and 2 NCRs (male) patients. PREX2 is a guanine nucleotide exchanger for RAC1. PREX2 dysfunction could result in RAC1 activation and elevated PI3K/AKT signaling which could result in higher cell proliferation (Lissanu Deribe 2016).

Variants in PREX2:

**Gene**	SNP	AA CHANGE	1000g_eas	1000g_all	ExAC_ALL	ExAC_EAS	SIFT	Polyphen2	CR number (n/14)	NCR number (n/15)
	rs141504768 (NC_000008.11: g.68087728G>T)	V678L	0.0923	0.024361	0.0125	0.0952	0.124,T	0.044,B	5	2
**PREX2**	rs61753703 (NC_000008.11: g.68146301C>T)	R1394W	0.0327	0.0129792	0.0086	0.0396	0.0,D	1.0,D	2	2
	rs61753704 (NC_000008.11: g.68192384C>T)	S1488L	0.1617	0.0609026	0.024	0.1701	0.038,D	0.999,D	4	1

Heterozygous for rs141504768:

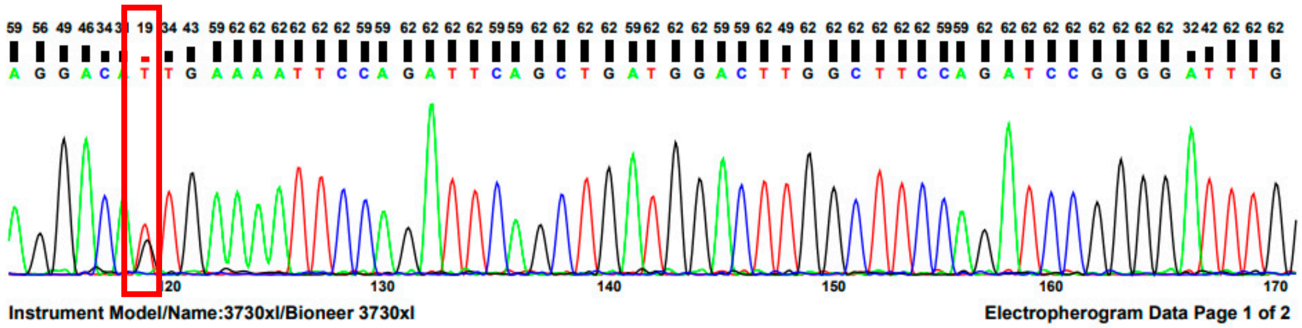

Heterozygous for rs61753703:

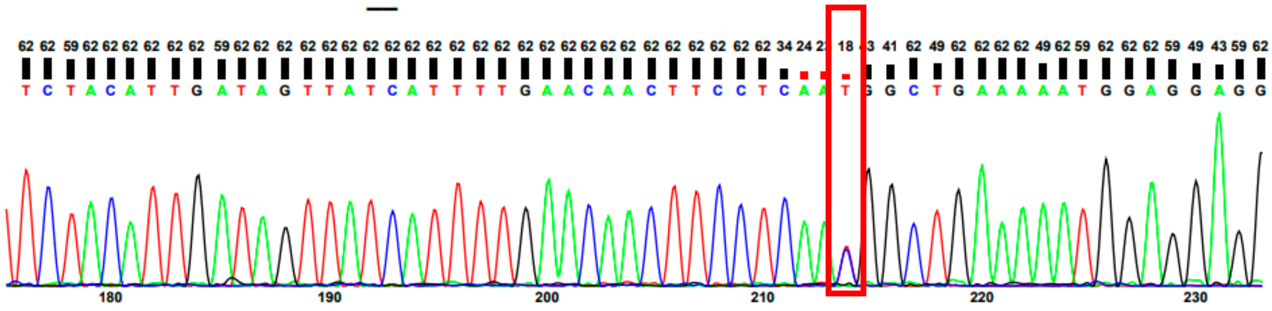

Common genes in CR and NCR

	CR														
	cr 1	cr 2	cr 3	cr 4	cr 6	cr 7	cr 8	cr 9	cr 10	cr 11	cr 12	cr 13	cr 14	cr 15	
Gene	1	2	3	4	5	6	7	8	9	#	#	#	#	#	Total (gene)
USP19	O	O	O	O	X	O	X	X	O	X	X	X	X	X	6
RPUSD3	X	X	X	O	X	X	O	X	X	X	O	X	O	O	5
PREX2	O	O	O	O	O	X	O	X	O	X	O	O	X	X	9
IRGM	O	O	O	O	O	O	O	O	O	X	X	X	O	X	10
TTLL3	X	O	O	O	O	O	O	O	X	X	X	O	X	O	9
HELT	O	O	X	X	X	O	O	O	O	X	X	O	O	X	8
FLCN	X	O	O	X	O	X	O	O	O	O	O	X	X	O	9
EDDM3B	X	O	X	O	O	O	X	X	O	X	O	O	O	X	8
WARS2	O	O	X	X	X	O	O	O	O	X	X	X	O	X	7
PDE4A	O	X	X	X	O	X	O	X	X	X	O	O	O	O	7
KIAA0895L	X	O	X	X	O	X	O	X	X	X	O	O	O	O	7
GLIS1	X	O	O	X	O	X	O	X	O	O	X	X	X	O	7
OR11A1	X	O	X	O	O	O	O	X	X	O	X	X	O	X	7
DCAF4	O	X	O	O	X	X	O	X	O	X	X	O	X	O	7
DHFRL1	O	O	X	X	X	O	O	X	X	O	O	O	X	X	7
C17orf96	X	X	X	O	X	O	O	X	X	O	O	O	X	O	7
ALDH5A1	O	O	X	O	O	X	X	O	X	X	O	X	O	X	7
ANTXR1	O	O	O	X	O	O	X	X	O	X	X	O	O	X	8
ACBD5	X	O	O	O	O	O	O	X	X	O	X	X	O	X	8
ELF1	O	O	X	O	O	O	X	O	O	O	X	X	O	O	10
DCTN4	O	O	X	X	O	O	O	O	O	O	O	X	O	X	10
NAV1	O	X	X	O	X	X	O	O	O	O	O	O	O	O	10
RAI14	O	X	O	O	X	O	O	O	O	O	O	O	O	X	11
CALML5	O	O	X	O	O	O	O	O	O	O	O	X	X	O	11
BRSK2	O	O	O	O	O	X	X	O	O	O	O	O	O	O	12

	NCR															
	ncr 1	ncr 2	ncr 3	ncr 9	ncr 10	ncr 11	ncr 12	ncr 13	ncr 14	ncr 15	ncr 16	ncr 17	ncr 18	mcr19	ncr 20	
Gene	1	2	3	4	5	6	7	8	9	10	11	12	13	14	15	Total (gene)
USP19	x	x	x	x	x	x	x	x	x	x	x	x	x	x	x	0
RPUSD3	x	x	x	x	x	x	x	x	x	x	x	x	x	x	x	0
PREX2	x	x	x	x	x		O	X	X	X	O	x	x	x	x	2
IRGM	x	x	x	x	x	O	X	X	X	O	X	x	x	x	x	2
TTLL3	x	x	x	x	x	x	x	X	X	X	X	O	O	x	x	2
HELT	x	x	x	x	x	x	x	O	X	X	X	x	x	x	x	1
FLCN	x	O	x	x	x	x	x	x	X	X	O	x	x	x	x	2
EDDM3B	x	O	X	O	x	x	x	x	X	X	x	x	x	x	x	2
WARS2	x	X	X	O	x	x	x	x	X	X	O	x	x	x	x	2
PDE4A	x	O	X	X	X	O	x	x	X	X	X	x	x	x	x	2
KIAA0895L	x	X	X	X	O	X	x	x	X	X	X	x	x	x	x	1
GLIS1	x	O	X	X	X	X	x	x	X	X	X	x	x	x	x	1
OR11A1	x	x	O	X	X	X	x	x	X	O	X	x	x	x	x	2
DCAF4	x	x	O	X	X	X	x	x	X	O	X	x	x	x	x	2
DHFRL1	x	x	O	X	X	O	x	x	X	X	X	x	x	x	x	2
C17orf96	x	x	X	X	O	X	x	x	X	X	X	O	x	x	x	2
ALDH5A1	x	O	X	X	O	X	x	x	O	X	X	X	x	x	x	3
ANTXR1	x	X	X	X	X	X	x	x	O	X	X	X	x	x	x	1
ACBD5	O	X	X	O	X	X	x	x	X	X	X	X	x	x	x	2
ELF1	X	X	X	X	X	X	X	O	O	X	O	X	x	x	O	4
DCTN4	X	X	X	X	X	O	X	X	X	O	X	X	x	O	X	3
NAV1	X	X	X	O	O	X	X	O	X	X	X	X	x	O	X	4
RAI14	O	X	O	X	X	X	X	X	O	X	X	X	x	O	X	4
CALML5	X	X	X	X	X	X	X	O	X	X	X	O	x	O	O	4
BRSK2	O	X	X	X	O	X	X	X	X	O	X	O	x	X	X	4

	CR														
	cr 1	cr 2	cr 3	cr 4	cr 6	cr 7	cr 8	cr 9	cr 10	cr 11	cr 12	cr 13	cr 14	cr 15	
IL3RA	x	x	O	x	x	x	x	x	x	x	x	x	x	x	1
AMER2	x	x	O	x	x	x	x	x	x	x	x	x	x	x	1
GPA33	X	x	O	X	X	X	x	x	x	x	x	x	X	X	1
ZNF546	X	x	X	O	X	O	x	x	x	x	x	x	X	X	2
PSMD5	X	x	X	X	X	O	x	x	x	x	x	x	O	X	2
CLEC3A	x	x	O	X	X	X	O	X	x	x	X	X	O	X	3
ZNF552	x	O	O	X	X	X	X	O	x	x	X	X	X	X	3
YDJC	X	X	X	X	O	X	X	X	x	x	X	X	O	O	3
SMG1	X	X	X	X	X		X	X	x	x	O	X	X	X	1
SAMD7	X	X	X	X	X	X	O		x	x	X	X	X	X	1
NCBP3	x	O	X	X	X	X	X	O	x	x	X	X	X	X	2
HPS1	O	X	X	X	O	X	X	X	x	x	X	X	X	X	2
TIMM21	X	X	X	X	X	X	X	X	x	x	X	O	X	O	2
RBP1	X	X	X	X	X	X	X	X	x	x	x	x	x	O	1
SLC4A1AP	X	X	X	X	O	O	X	X	x	x	x	x	x	X	2
PRPS1L1	X	X	X	X	O	X	X	X	O	x	x	x	x	x	2
PSG8	X	X	X	X	O	X	X	X	X	x	x	x	x	x	1
PLSCR4	X	O	O	X	X	X	X	X	X	O	x	x	x	x	3
PRKRA	X	X	X	X	X	X	X	X	X	X	O	X	O	X	2
LRRC8B	X	X	X	O	X	X	X	X	X	X	X	X	X	X	1
LY6G5B	X	X	X	X	X	X	X	X	X	X	O	O	X	X	2
OR5I1	O	X	X	X	O	X	X	X	X	X	X	X	X	X	2
OR7D4	X	X	O	X	X	X	X	X	X	X	X	X	O	X	2
NUMA1	X	X	X	X	X	X	X	O	X	X	X	X	X	X	1
FILIP1L	X	X	O	X	X	O	X	X	X	X	X	X	X	X	2
ERC1	X	X	X	O	X	X	X	X	O	X	X	X	X	X	2
CPD	O	X	X	X	X	X	X	X	X	X	X	X	X	X	1
C2orf61	X	O	X	X	X	X	X	X	X	X	X	X	X	X	1
CD8B	X	X	O	O	O	X	X	X	X	X	X	X	X	X	3
CNKSR1	X	X	O	X	O	X	X	X	O	X	X	X	X	X	3
AVL9	X	X	O	X	X	X	X	O	X	X	X	O	X	X	3
ACD	X	X	O	X	O	X	X	X	O	X	X	X	X	X	3
SETD1B	X	X	X	X	X	X	X	X	X	O	O	X	X	X	2
FAAH	X	X	X	X	X	O	X	X	O	X	X	O	X	X	3
AMPD2	O	X	O	X	X	X	X	O	O	X	X	X	X	X	4
CUL3	X	X	X	X	O	X	O	X	X	X	X	X	O	X	3
PIH1D1	O	O	O	O	X	X	X	X	X	X	X	X	X	X	4
FCRL3	X	O	X	O	X	X	O	X	X	O	O	X	X	X	5

	NCR															
	ncr 1	ncr 2	ncr 3	ncr 9	ncr 10	ncr 11	ncr 12	ncr 13	ncr 14	ncr 15	ncr 16	ncr 17	ncr 18	mcr19	ncr 20	
IL3RA	X	O	O	O	X	O	X	X	X	X	X	O	X	X	X	5
AMER2	O	O	X	X	O	X	X	X	X	X	X	O	O	X	X	5
GPA33	O	O	O	O	X	X	O	X	X	O	X	X	O	X	X	7
ZNF546	O	O	X	X	X	O	X	O	O	O	O	O	O	X	X	9
PSMD5	X	X	O	O	O	O	X	O	X	X	O	O	X	O	O	9
CLEC3A	X	O	X	X	X	O	O	O	O	O	O	X	X	O	O	9
ZNF552	O	O	X	X	O	O	X	O	X	O	O	X	O	X	X	8
YDJC	X	X	O	O	X	X	O	O	O	X	O	O	X	O	X	8
SMG1	O	O	X	X	O	X	X	O	O	X	X	X	O	O	O	8
SAMD7	O	O	X	X	O	O	X	X	X	X	O	O	O	O	X	8
NCBP3	O	O	X	X	X	X	O	O	X	O	O	X	X	O	O	8
HPS1	X	O	O	X	X	X	O	X	O	O	O	O	X	X	O	8
TIMM21	O	O	X	X	O	O	X	X	O	X	O	O	X	X	X	7
RBP1	O	X	O	X	O	X	X	X	O	X	O	O	X	X	O	7
SLC4A1AP	X	X	X	O	X	O	X	O	X	X	O	O	O	X	O	7
PRPS1L1	X	O	X	O	X	X	X	X	X	O	O	X	O	O	O	7
PSG8	O	X	O	O	O	X	X	X	X	O	O	X	O	X	X	7
PLSCR4	O	X	O	X	X		X	O	X	O	O	X	O	X	O	7
PRKRA	O	X	X	X	O	O	O	O	X	X	X	X	O	X	O	7
LRRC8B	O	O	X	X	X	O	X	X	X	O	X	O	O	O	X	7
LY6G5B	X	O	X	O	O	O	O	X	X	O	O	X	X	X	X	7
OR5I1	O	X	X	X	O	X	O	O	O	O	X	X	X	O	X	7
OR7D4	O	X	O	O	O	X	O	X	X	X	O	X	X	O	X	7
NUMA1	X	X	X	X	O	X	O	O	O	X	X	O	O	X	O	7
FILIP1L	O	O	X	X	X	X	O	O	O	O	X	X	O	X	X	7
ERC1	O	X	X	X	O	O	O	O	O	X	X	X	O	X	X	7
CPD	O	O	X	X	X	O	O	X	X	X	O	O	O	X	X	7
C2orf61	X	X	O	X	O	O	X	X	X	O	X	O	O	O	X	7
CD8B	O	X	X	O	X	X	O	O	X	O	O	X	X	X	O	7
CNKSR1	X	X	X	O	X	X	X	X	O	O	X	O	O	O	O	7
AVL9	X	O	O	X	X	X	O	O	O	X	O	X	X	O	X	7
ACD	X	X	O	O	O	X	X	X	X	O	O	O	O	X	X	7
SETD1B	X	X	X	O	O	O	X	O	O	O	X	O	O	O	O	10
FAAH	X	O	O	O	O	X	O	O	O	X	O	X	O	O	X	10
AMPD2	X	O	X	X	O	O	X	O	O	O	X	O	O	O	O	10
CUL3	O	X	O	O	O	X	O	O	O	X	O	X	O	O	O	11
PIH1D1	O	O	O	O	O	O	X	X	O	O	O	O	O	X	O	12
FCRL3	O	O	O	O	O	O	X	O	O	O	O	O	O	O	O	14
